# A 22 Gene-expression Assay, Decipher® (GenomeDx Biosciences) to Predict Five-year Risk of Metastatic Prostate Cancer in Men Treated with Radical Prostatectomy

**DOI:** 10.1371/currents.eogt.761b81608129ed61b0b48d42c04f92a4

**Published:** 2015-11-17

**Authors:** Michael Marrone, Arnold L. Potosky, David Penson, Andrew N. Freedman

**Affiliations:** Epidemiology and Genomics Research Program, Division of Cancer Control and Population Sciences, National Cancer Institute, NIH, Rockville, Maryland, USA; Lombardi Comprehensive Cancer Center, Georgetown University Medical Center, Washington DC, USA; Department of Urology, Vanderbilt University Medical Center, Nashville, Tennessee, USA; Epidemiology and Genomics Research Program, Division of Cancer Control and Population Sciences, National Cancer Institute, NIH, Rockville, Maryland, USA

## Abstract

Among the estimated 230,000 men diagnosed with prostate cancer in the US each year there has been a rise in the number of radical prostatectomies (RP). There is some debate over the value of immediate adjuvant therapy following RP in men with high-risk pathological features versus delayed salvage radiation therapy when signs of disease progression are observed. Thus, it would be potentially useful to inform post-RP management strategies by more clearly identifying those patients at higher risk of progression and death from prostate cancer. A 22 gene-expression assay, Decipher® (GenomeDx Biosciences), has been developed in men treated with radical prostatectomy to predict the five-year risk of metastatic prostate cancer. Published and unpublished literature was evaluated to determine the analytic validity, clinical validity and clinical utility of Decipher. Limited information is available on the analytic validity of Decipher. In both discovery and validation studies, Decipher was shown to have good performance in discriminating men with metastasis from men without metastasis five years after surgery (AUC 0.75 to 0.90). In terms of clinical utility, no evidence was found reporting improved outcomes (lower prostate cancer specific mortality and treatment related adverse effects) from using this test to guide post-operative treatment. Four studies provided weak indirect evidence of clinical utility in which 31% to 43% of post-operative treatment recommendations were changed in men with high-risk prostate cancer based on test results, with 27% to 52% of treatment recommendations changing from any treatment to no treatment.

## Clinical scenario

In men diagnosed with prostate cancer in the US, prognosis is generally excellent following radical prostatectomy (RP) with 15-year disease free survival of 93%.^1^ However, RP may be associated with significant complications and adverse-effects, primarily sexual dysfunction and urinary incontinence. Furthermore, between 19% and 30% of men experience biochemical recurrence (rise in PSA over 0.2 ng/ml) 5 to 10 years after surgery, and these men have a 37% risk of developing distant metastasis within five years if not treated and have a 17% risk of dying from prostate cancer within six years of biochemical recurrence.^2^^,^ 3 The most appropriate course of treatment is based on standard risk assessment that incorporates clinical characteristics such as cancer stage, grade, and size as well as histopathological features including PSA and Gleason score to estimate risk of recurrence from nomograms.^4^ Current post-RP practice includes observation for low risk disease, adjuvant therapy for adverse pathology and salvage therapy after biochemical recurrence.^4^ However, not all men with high-risk prostate cancer with adverse pathological features will require or will benefit from adjuvant therapy. Furthermore, the adverse events and complications associated with postoperative adjuvant therapy are not insignificant and range from incontinence, bowel complications as well as urogenital toxicities resulting from radiotherapy.^5^ Thus, there remains a need for increased accuracy in estimating a man's prognosis (risk of metastasis) to ensure appropriate follow up care is given in a timely manner.

## Test Description

Decipher® (GenomeDx Biosciences) is a genomic test that was developed to predict the risk of metastatic prostate cancer within five years of RP in men at high risk of recurrence (extraprostatic extension, seminal vesicle invasion, positive margins, or biochemical recurrence).^6^ A genomic classifier score calculated from a gene-expression microarray analysis of 22 genes on formalin-fixed, paraffin embedded (FFPE) prostate tumor tissue ranges between 0 and 1 and classifies patients as high risk (1 in 5 risk of metastasis), average risk, or low risk (1 in 42 risk of metastasis).^6 ^No information was available on the specific genes included in the 22-geen analysis. Decipher is available as a laboratory developed test through physicians or as part of clinical investigations sponsored by GenomDx, which is licensed under the Clinical Laboratory Improvement Act (CLIA).

## Public Health Importance

In the US, prostate cancer is the most common cancer in men with estimates showing over 230,000 cases will be diagnosed in 2014 and just under 30,000 men will die from the disease.^7^ For men with high-risk prostate cancer based on post-RP pathological findings the strongest predictors of prostate cancer metastasis and death are baseline Gleason score, time of biochemical recurrence after RP, and PSA doubling-time.^2^^,^ 3 Within this high-risk group of men with prostate cancer the benefit of adjuvant or salvage therapy varies, and the high costs and complications associated with adjuvant radiotherapy underscore the need for improved prognostic markers.^5^

## Methods

PubMed was searched (14 July 2015) to identify published reports of studies investigating the analytic validity, clinical validity and clinical utility of Decipher. Supplemental searches for grey literature, guidelines, and coverage decisions included Google, Genetic Testing Registry (GTR), guidelines.gov, and the Centers for Medicare and Medicaid using the terms “Decipher genomic classifier” and “Decipher”. Websites for Decipher^6^ and GenomeDx^8^ were also reviewed to identified additional published and unpublished information. No trial registries were searched, which is a possible limitation.

PubMed search strategy:

((classifier[tiab]) AND ((genomic*[tiab]) OR (genome*[tiab]))) AND ((prostate[tiab] OR prostatic[tiab] OR (prostatic neoplasm[mesh]) OR (biochemical recurrence[tiab]))

**Published Reviews, Recommendations, and Guidelines**

No published reviews, recommendations or guidelines describing the Decipher genomic classifier were identified from the methods described above. One local coverage decision from Palmetto GBA was identified providing limited coverage for Decipher in order to determine which patients at high-risk of recurrence following RP should receive radiation therapy vs. continued observation.^9^

**Analytic Validity**

Data reported in a conference abstract^10^ showed RNA was successfully extracted from 91% (63/69) FFPE biopsies and 100% (69/69) of RP tissue samples, and 98% (62/63) and 99% (68/69) of the FFPE and RP samples passed gene-expression data quality control, respectively. In this study, the correlation between matched biopsy FFPE tissue and RP tissue samples in the genomic classifier scores predicting metastasis was 0.74 (p=0.0003).

Two other studies are listed on the Decipher website under ‘Analytic Validity’,^11^^,^ 12 but it is unclear whether the methods and procedures described in these reports are integrated into the Decipher analytic pipeline.

**Clinical Validity**

Eight published studies were identified evaluating the clinical validity of the genomic classifier, based on its ability to predict clinical metastasis^13^^,^ 14 ^,^ 15 ^,^ 16 ^,^ 17 ^,^ 18 ^,^ 19 ^,^ 20. The first study used data from a Mayo Clinic patient registry to compare men with evidence of clinical metastasis following RP (cases) to men with no evidence of metastasis after RP (controls) with median follow-up greater than five years. One publication reported results from the initial training and validation datasets in which participants with no evidence of disease recurrence and those with biochemical recurrence (PSA > 0.20 ng/ml within 30 days of RP) served as controls due to “very limited differential expression” between these two groups.^16^ Therefore, the genomic classifier was developed to discriminate men who develop clinical metastasis from men who have no evidence of disease recurrence (clinical or biochemical) within five years of RP. Two additional studies using data from the Mayo Clinic registry described subsequent validation of the genomic classifier in men with biochemical recurrence after RP^19^ and in men with high-risk prostate cancer (pre-RP PSA > 20ng/mL, Gleason score > 8, pT3b, or Mayo Clinic nomogram score > 10).^17^

Overall, the genomic classifier had good discriminatory ability (e.g. ability to predict clinical metastasis) in both the discovery and validation studies (Table 1). Across the nine analyses, the area under the receiver operating curve (AUC) ranged between 0.75 and 0.90. None of the studies examining the predictive accuracy of the genomic classifier explicitly reported whether the gene-expression analysis was performed on tissue collected at the time of surgery or at the time of clinical end point (e.g. metastasis). There was potential overlap in participants used to develop and validate the genomic classifier across four studies interrogating a patient registry^13^^,^ 16 ^,^ 17 ^,^ 19. In three full-text validation studies^16^^,^ 17 ^,^ 19 there were noticeable differences between cases and controls in the frequency of high-risk pathological features, risk factors for metastasis, and adjuvant and salvage therapy (Table 2). The possibility of confounding due to these differences may have yielded an overestimate of the prognostic ability of the genomic classifier. Another limitation is the change in risk of metastasis with more follow up time. The 5-year risk of metastasis among men with biochemical recurrence is only 4%, whereas the 10- and 15-year risk increases dramatically (11% and 19%)^21^, suggesting the genomic classifier may lead to false-negative results for those men who experience metastasis beyond five years. In terms of prostate cancer-specific mortality, one study reported an AUC of 0.78 (95% CI: 0.68 to 0.87) among high-risk patients^13^. In a group of men treated with adjuvant radiation therapy, the AUC for metastasis was 0.83 (95% CI: 0.27 to 0.89).^15^

A conference abstract^22^ reported that the genomic classifier could predict metastasis in a subset of prostate cancer tumors with ETS gene overexpression, PTEN-loss, or both very well. Among the tumors with ETS gene overexpression and PTEN loss the genomic classifier had an AUC of 0.82 and 0.85, respectively. In tumors with both ETS overexpression and PTEN loss, the AUC was 0.85.

The cumulative incidence of metastasis five years after RP was 2.4%, 6.0%, and 22.5% for the low, intermediate, and high risk groups defined by the genomic classifier respectively^17^ and 0%, 12%, 17% eight years after RP^14^ and 12%, 31% and 47% 10 years after RP for the same three risk groups respectively^20^.

**Figure:**
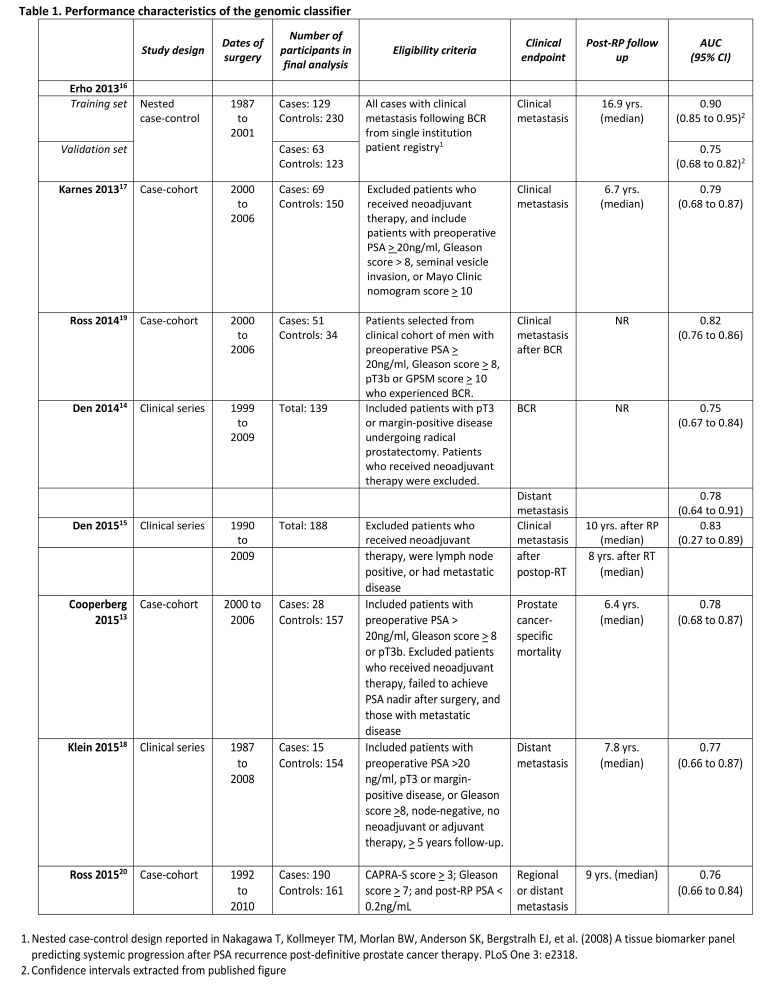
Table 1: Performance characteristics of the genomic classifier

**Figure:**
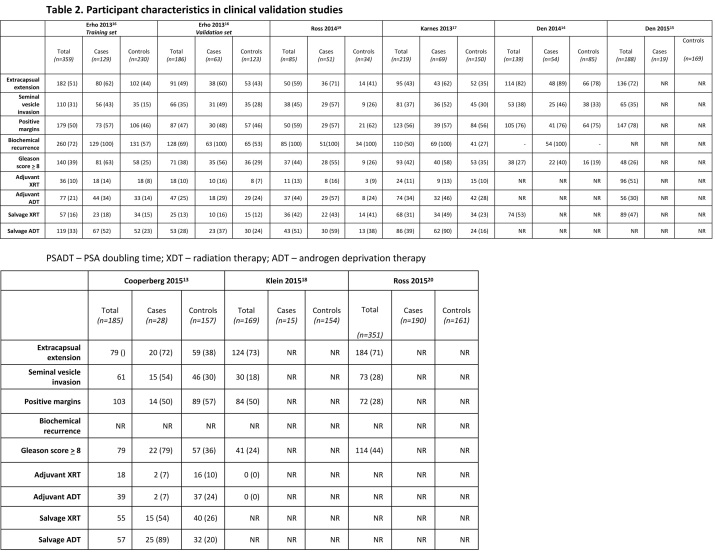
Table 2: Participant characteristics in clinical validation studies

**Clinical Utility**

No direct evidence for clinical utility for the genomic classifier is currently available showing changes in selection of primary or adjuvant treatment based on the genomic classifier or showing that the genomic classifier can successfully predict treatment outcomes. There is only limited evidence of clinical utility from four publications^23^^,^ 24 ^,^ 25 ^,^ 26 and one conference abstract^27^ comparing physician’s treatment recommendations before and after knowledge of the results from the genomic classifier. Study investigators recruited physicians to review the medical records of selected patients from an actual practice with high-risk prostate cancer in the adjuvant setting (no evidence of biochemical recurrence after RP) and salvage setting (evidence of biochemical recurrence after RP). Physicians were asked to make treatment recommendations based on their reviews. The overall change in treatment recommendations, after asking physicians to consider the scores from the genomic classifier, ranged between 31% and 43% in the adjuvant setting and 53% in the salvage setting (Table 3). The change from no treatment (observation) to any therapy ranged from 8% to 38%, and from any treatment to no treatment ranged from 27% to 52%. All study reports included case histories of high-risk prostate cancer in which the most significant change in treatment recommendations was from any adjuvant therapy to no treatment. These studies provide indirect evidence that only suggests some potential for the genomic classifier to achieve clinical utility.

**Figure:**
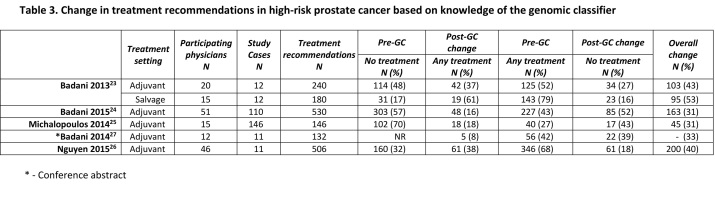
Table 3: Change in treatment recommendation in high-risk prostate cancer based on knowledge of the genomic classifier

## Conclusions

The recently introduced genomic classifier was developed to identify men with increased risk for metastatic prostate cancer following radical prostatectomy. The genomic classifier was shown to have good discrimination in detecting men at risk for metastatic prostate cancer five years after surgery (AUC 0.75 to 0.90). However, these estimates may be biased given differences in important prognostic characteristics between cases and controls. Independent validation in patients with high-risk prostate cancer may provide more reliable estimates of the prognostic ability of the genomic classifier. At this time it is unclear what the most appropriate clinical actions are based on test results. No studies yet published have reported the ability of the genomic classifier to predict significant clinical outcomes from any of the available adjuvant therapies. In order to demonstrate clinical utility additional evidence is needed showing improved outcomes (clinical metastasis or prostate cancer-specific mortality) in men whose post-operative treatment was guided by the genomic classifier compared to standard practice.

## Competing Interests

The authors have declared that no competing interests exist. The findings and conclusions are those of authors, including NIH co-authors, and do not necessarily represent the views of the National Institutes of Health (NIH). The information provided in this manuscript does not constitute an endorsement of any of the commercially available tests described in this report by NIH nor the Department of Health and Human Services of the U.S. government.
